# Gait, physical function, and physical activity in three groups of home-dwelling older adults with different severity of cognitive impairment – a cross-sectional study

**DOI:** 10.1186/s12877-021-02598-9

**Published:** 2021-12-01

**Authors:** Kristin Taraldsen, Jorunn L. Helbostad, Turid Follestad, Sverre Bergh, Geir Selbæk, Ingvild Saltvedt

**Affiliations:** 1 Department of Physiotherapy, Faculty of Health Sciences, Oslo Metropolitan University (OsloMet), Oslo, Norway; 2 Department of Neuromedicine and Movement Science, Faculty of Medicine and Health Sciences, Norwegian University of Science and Technology (NTNU), Trondheim, Norway; 3 Clinic of Clinical Service, St Olav University Hospital, Trondheim, Norway; 4 Department of Clinical and Molecular Medicine, Faculty of Medicine and Health Sciences, Norwegian University of Science and Technology (NTNU), Trondheim, Norway; 5 Research Centre for Age-related Functional Decline and Disease, Innlandet Hospital Trust, Ottestad, Norway; 6 Norwegian National Advisory Unit on Ageing and Health, Vestfold Hospital Trust, Tønsberg, Norway; 7 Department of Geriatric Medicine, Oslo University Hospital, Oslo, Norway; 8 Faculty of Medicine, University of Oslo, Oslo, Norway; 9 Department of Geriatrics, St Olav University Hospital, Trondheim, Norway

**Keywords:** Elderly, Physical activity, Accelerometers, Dementia

## Abstract

**Background:**

The research on associations between gait, physical function, physical activity (PA), and cognitive function is growing. Still, clinical assessments of cognitive function and motor function is often kept separate. In this study, we aimed to look at a broad range of measures of gait, physical function, and PA in three groups of home-dwelling older adults with no or questionable dementia, mild dementia, and moderate/severe dementia.

**Methods:**

This cross-sectional study included 100 home-dwelling older adults, recruited from an outpatient geriatric memory clinic. Severity of dementia was categorised using the clinical dementia rating scale (CDR), with no or questionable dementia (CDR score 0 and 0.5), mild dementia (CDR score 1) and moderate/severe dementia (CDR score 2 and 3). We used thigh worn accelerometers to measure daily PA, the Short Physical Performance Battery (SPPB) to measure physical function, and an electronic gait mat to evaluate gait characteristics. Associations between severity of dementia and measures of PA, physical function, and gait characteristics were assessed by linear regression.

**Results:**

Participants’ (mean age 78.9 (SD 6.7) years, 57% women) average gait speed was 0.93 m/sec, and average upright time was 301 min/day. Statistically significant associations were found for the severity of dementia and gait speed (*p*=0.002), step time (*p*=0.001), physical function (SPPB, *p*=0.007), and PA (upright time, *p*=0.031), after adjusting for age. Overall, having no or questionable dementia was associated with faster gait speed (mean difference 0.163 (95% CI: 0.053 to 0.273)), shorter step time (-0.043 (-0.082 to -0.005)), better SPPB score (1.7 (0.5 to 2.8)), and longer upright time (78.9 (18.9 to 139.0)), compared to those with mild dementia. Furthermore, having no or questionable dementia was also associated with faster gait speed and better SPPB scores, as compared to those with moderate to severe dementia. No evidence of any differences was found between the participants with the mild dementia versus the moderate to severe dementia.

**Conclusions:**

After adjusting for age, we found that the no or questionable dementia group to be associated with better gait and physical function, and more PA, as compared with the two groups with mild or moderate/severe dementia. Evaluation of gait, physical function, and PA can add clinically important information of everyday functioning in memory clinics meeting geriatric patients, but investigations on how to use these results to guide interventions are still needed.

## Background

It is well known that gait and physical function is affected among patients with advanced dementia. Slow gait speed may also precede decline in cognitive function in healthy older adults [1]. Furthermore, gait impairment in older persons is linked to cognitive decline, in particular executive function [2]. There is also evidence that the combination of slow gait and cognitive complaints may identify individuals at high risk for developing dementia [3–5]. In fact, regardless of the definition used, gait performance and cognitive dysfunction is suggested closely related, as poor gait performance has been shown to be present years before dementia was diagnosed [6]. It is suggested that research on the interrelationship between cognitive functioning and gait is important for understanding how the two are related and potentially help targeting interventions in order to prevent functional decline and improve quality of life at older age [7].

Level of Physical activity (PA) is associated with physical function at old age, and physical activity is suggested to be beneficial to maintain cognitive function [8]. For persons with established cognitive impairment, important benefits of PA, such as maintaining physical function and ability to perform everyday-life functions, have been demonstrated [9, 10]. A recent publication from a longitudinal study also found an association between physical activity and slower cognitive decline among participants with both low and high concentrations of total tau, a brain protein used as a blood biomarker for Alzheimer’s disease [11]. Objective measures of PA are however rarely included in assessment of persons with cognitive impairment, and how physically active these persons actually are, is not well described.

Based on the literature it is reason to believe that even persons with mild cognitive impairment (MCI) have alterations in gait characteristics [3] that will be increasing with the severity of dementia. Gait function and PA measures could potentially provide useful information when suspecting a cognitive impairment, but such measures are more rarely included in clinical evaluations. In this exploratory study, we aimed to examine gait, physical function, and PA, in three groups of different severity of cognitive impairment, in persons at the time of the first clinical work-up at a geriatric memory clinic.

## Methods

### Study design and setting

This is an observational, cross-sectional study performed at the Geriatric Memory Clinic at St Olavs University Hospital, Trondheim, Norway, where older patients with potential symptoms of cognitive impairment are referred from general practitioners. Participants were eligible for participation if they had agreed to take part in the Norwegian Register of Persons assessed for Cognitive Symptoms (NorCog) and were able to give their informed consent. The study was approved by the Norwegian Ethical Committee (2014/1689/REK sør-øst A).

### Participants

Participants were included from February 2015 to April 2016. Consecutive inclusion at the outpatient clinic was performed until 100 participants were included. Inclusion criteria were being a participant in the NorCog register, living at home, living in the municipality of Trondheim, and being able to walk.

### Procedure

The clinical assessment of the participants was performed in accordance with international and national guidelines [12] by use of assessments for the NorCog register completed by consultants and nurses employed at the memory clinic. Patients and caregivers were interviewed about symptoms and function, and all patients underwent clinical examination and cognitive testing.

Physical examination, lasting 15 to 30 minutes, was performed by a physiotherapist in a gait lab immediately after the standard cognitive work up. An activity monitor was fixed to the participants’ thigh and was worn continuously for one week.

### Background information

We collected information about body height and weight, age, gender, marital status, years of education, occupational status, living arrangements, whether people lived alone or not, and whether they received assistance from the municipality health care service.

Instrumental Activities of Daily Living (IADL) was assessed with the Lawton and Brody (1969) scale [13]. The scale comprises assessment of need of assistance in eight instrumental activities of daily living: using the phone, grocery shopping, preparing meals, housekeeping, laundering, using transportation, taking medications, and managing finances, and for each item the responders scored according to their highest functional level. A summary-score was calculated, ranging from 0 to 8 for women and 0 to 5 for men [14, 15], from low (dependent) to high (independent) function. Single missing items were given the score 0. Grip strength (in kg) on dominant side, was measured by use of a hand-held dynamometer (JAMAR) by using the best score out of three.

### Measurements of cognitive function

The Clinical Dementia Rating scale (CDR) was used to rate the severity of dementia [16]. The medical doctor scored the CDR after completing a broader test battery of cognitive tests, and the scale represents how cognitive function affect aspects in their daily life. In the present study, we created three groups based on the CDR scores of 0 and 0.5 (no or questionable dementia), 1 (mild dementia), and 2 and 3 (moderate and severe dementia).

To have descriptive in-depth information about cognitive function we also included additional performance-based tests. The Norwegian version of the Mini Mental State Examination (MMSE) was used to describe global cognitive function [17]. The test is scored from 0 to 30, with 30 as the best score. The Clock Drawing Test (CDT) [18] was used as a measure of visuospatial abilities and executive function, with test scores ranged from 0 (inability to make any reasonable attempt) to 5 (perfect clock) [19]. The Trail Making Test A and B (TMT-A and TMT-B) [20–22] were used to assess psychomotor speed, attention, and executive function. The time to complete TMT-A and TMT-B was registered (seconds), where long time reveal greater impairment. For participants able to start but not completing the tests within 300 seconds for TMT-A and 180 seconds for TMT-B, the tests’ results were set to maximum time [23, 24].

### Gait measurements

Gait characteristics were assessed by use of an electronic gait mat, GAITRite® mat (CIR systems Inc. Sparta, US), with a 4.88 m active area in the middle of an 8.0 m walkway. Participants walked the gait mat back and forth at an instructed preferred gait speed with their usual walking aids, if needed. If two walking trials were available, the mean value from the trials was calculated. Data were processed using the PKmas® software, where the mean and within-subject standard deviation (SD) of the steps within a walk, and the left/right ratio of spatial and temporal gait variables were calculated by the software. Gait speed is a robust measure of health and function [25]. In addition to gait speed (m/s), we used the following gait variables: step time (s), walk ratio (step length/cadence, where cadence is steps/minute), and the variability measure SD stride velocity (cm/sec), selected as potential metrics relevant for postural control during gait [26].

### Physical function measurements

We used the Short Physical Performance Battery (SPPB) [27] as a measure of physical function. SPPB consists of three items, standing balance in three different positions, 4-meter walking, and five chair stands, and each item scored on a scale from 0 to 4. The total score on SPPB ranges from 0 to 12, with 12 the best score.

### Physical activity measurements

We used small body worn accelerometers from PAL Technologies (Glasgow, UK) to monitor daily PA. The accelerometer was attached to the participants’ right thigh and was measured acceleration continuously until removal of the sensor after one week. We included data from participants with a minimum of one day (24 hours) of complete recordings in the analyses. Mean time in upright position per day (Upright time) and mean number of sit-to-stand transitions per day (Upright events) were calculated from the data and used as outcome measures, along with information about the maximum length of the upright events (Max Event Length). The activity monitor and algorithms have been validated in a sample of older adults, showing valid measures of upright time and upright events [28]. Max Event Length is a more exploratory metric, but previously shown to be a predictor for outdoor mobility in hip fracture patients [29]. Multiple recording days for PA measures are recommended, however when comparing on a group level, one day of PA recordings has shown to be sufficient in a sample of hip fracture patients [30].

### Statistical Analysis

The data are described as mean (SD) or percentages, as appropriate. Linear regression analyses were performed to examine the associations between dementia severity (CDR groups) and PA, physical function, and gait. Separate regression models were fitted for the dependent variables upright time, upright events, max event length, SPPB, gait speed, step time, walk ratio, and stride velocity variability. The linear regression models included CDR group, age and their interaction as explanatory variables. Residuals were checked for normality by visual inspection of normal Q-Q-plots. The significance level was set to 0.05. No formal adjustment of multiple testing was included. The results are presented as estimated mean differences with 95% confidence intervals and p-values. All statistical analyses were performed using IBM SPSS 24 software (SPSS Inc., Chicago, IL, USA).

## Results

We included a total of 100 participants with age ranging from 64 to 93 years (57 women). In total, 60% of the 100 participants lived together with their spouse/others and 38% reported that they had assistance from municipal home care services. For our analysis, according to the CDR scores, 38% of the participants were classified as “no or questionable dementia” (group 1), 39% as “mild dementia” (group 2), and 23% as “moderate to severe dementia” (group 3). Participant descriptive information is presented in Table 1.

**Table 1:** Demographic and clinical characteristics, cognitive function of participants in the three groups

	No or questionable dementia Group 1 (***n***=38)		Mild dementia Group 2 (***n***=39)		Moderate to severe dementia Group 3 (***n***=23)		Group differences
Characteristics		no. (%)		no. (%)		no. (%)	
Women		20 (52.6)		24 (61.5)		13 (56.5)	
Characteristics	n	Mean (SD, min-max)	n	Mean (SD, min-max)	n	Mean (SD, min-max)	***P***-values
Age, years	38	76.7 (6.7, 64-88)	39	79.9 (6.2, 65-92)	23	80.9 (6.8, 66-93)	0.032
BMI	32	25.4 (4.4, 16.3-33.1)	32	25.4 (3.9, 19.4-34.8)	22	24.9 (3.9, 17.5-32.2)	0.885
Years of education	36	11.1 (3.4, 7-21)	39	10.5 (3.1, 7-22)	22	9.8 (3.1, 7-17)	0.364
IADL score, 0-8, women	16	6.9 (1.4, 4-8)	20	5.8 (1.1, 4-8)	12	2.6 (1.4, 0-5)	<0.001
IADL score, 0-5, men	16	4.1 (0.8, 3-5)	14	3.3 (1.1, 1-5)	8	2.3 (0.7, 1-3)	<0.001
Medications used on regular basis, no.	36	4.5 (2.6, 0-11)	35	4.7 (3.1, 0-12)	23		0.710
Grip strength, kg WOMEN	16	21.6 (8.9, 12-49)	19	18.2 (4.0, 9-25)	9	16.0 (4.4, 8-20)	0.086
Grip strength, kg, MEN	14	35.7 (9.4, 24-56)	9	26.4 (7.0, 18-42)	9	29.2 (8.2, 20-42)	0.039
MMSE, score, 0-30	36	25.0 (4.1, 10-30)	34	21.9 (4.2, 15-30)	23	17.0 (3.9, 10-25)	<0.001
CDT, score, 0-5	36	3.8 (1.4, 0-5)	35	3.7 (1.4, 0-5)	22	2.1 (1.1, 0-4)	<0.001
TMT-A time, sec^a^	38	89.5 (68.8, 23-300)	38	138.0 (89.5, 42-300)	20	170.5 (88.7, 52-300)	0.001
TMT-B time, sec^b^	34	164.2 (32.7, 48-180)	32	170.9 (22.0, 105-180)	12	180	0.173

Groups are based on the CDR score where group 1 has a CDR score of 0 or 0.5, group 2 has a CDR score of 1, and group 3 has a CDR score of 2 and 3; *SD* Standard Deviation, *BMI* Body Mass Index, *IADL* Instrumental Activities of Daily Living, *MMSE* Mini Mental State Examination, *CDT* Clock Drawing Test, *TMT-A* The Trail Making Test A, *TMT-B* The Trail Making Test B. ^a^For the TMT-A maximum second for test was set to 300 seconds; ^b^ For the TMT-B maximum second for test was set to 180 seconds. The one-way analysis of variance (ANOVA) was used to indicate any statistically significant differences between the three groups.

The 88 participants fulfilling the criterion of at least 24 hours of complete recordings, wore the activity monitor for on average 4.2 days (SD 1.98), with a minimum of 48 hours for five participants. Participants spent on average 301 minutes per day in an upright position (standing and walking). Most of the participants walked without any walking aids (86%), while 5% walked with a roller, and 9% walked with one or two canes/crutches. There was no difference between groups in walking with or without walking aids during testing (*p*=0.950). Mean gait speed was 0.93 m/sec. Descriptive information of participants’ gait characteristics, physical function and PA is presented in Table 2.

**Table 2:** Gait characteristics, physical function and physical activity of participants in the three groups

	No or questionable dementia Group 1 (***n***=38)		Mild dementia Group 2 (***n***=39)		Moderate to severe dementia Group 3 (***n***=23)	
Characteristics:	n	Mean (SD, min-max)	n	Mean (SD, min-max)	n	Mean (SD, min-max)
**Gait**						
Gait speed, m/s	34	1.10 (0.24, 0.52-1.49)	33	0.91 (0.21, 0.44-1.23)	18	0.87 (0.23, 0.43-1.18)
Step time, s	34	0.58 (0.7, 0.45-0.78)	33	0.61 (0.1, 0.51-0.95)	18	0.62 (0.9, 0.49-0.83)
Walk ratio, SL/Cad	34	0.6 (0.1, 0.3-0.9)	33	0.5 (0.1, 0.4-0.8)	18	0.5 (0.1, 0.3-0.8)
SD stride velocity, cm/s	34	3.86 (1.2, 2.10-7.22)	33	4.13 (1.4, 2.01-6.96)	18	4.0 (0.94, 2.45-5.83)
**Physical function**						
SPPB score, 0-12	35	10.4 (2.1, 5-12)	37	8.3 (2.7, 3-12)	20	8.1 (2.8, 3-12)
**Physical activity**						
Upright time, minutes	35	345.7 (97.3, 148.9-637.4)	35	265.7 (125.6, 95.3-572.2)	18	282.4 (160.7, 74.1-648.1)
Upright events, number	35	48.8 (15.5, 26.3-92.0)	35	45.9 (16.5, 23.2-91.0)	18	45.2 (12.7, 26.3-70.0)
Length of upright events, max	35	83.6 (38.6, 28.4-213.0)	35	68.3 (47.2, 14.0-228.2)	18	67.0 (36.6, 11.4-129.2)

Groups are based on the CDR score where group 1 has a CDR score of 0 or 0.5, group 2 has a CDR score of 1, and group 3 has a CDR score of 2 and 3; Physical activity was measured over a period of 2 to 7 days in this sample. *SD* Standard Deviation, *SPPB* The Short Physical Performance Battery

The interaction effect between age and group was found not to be statistically significant in seven of eight linear regression models, with exception for gait speed (*p*=0.021), therefore results from linear regression with main effects only are presented. After adjusting for age, there was evidence in the data, to different degrees, that cognitive function was associated with gait speed (*p*=0.002), step time (*p*=0.001), SPPB (*p*=0.007), and upright time (*p*=0.031), see Table 3). Overall, the “no or questionable dementia” group differed compared to the two other groups while here was little or no evidence in the data for differences between the mild versus the moderate to severe dementia groups. Those with “no or questionable dementia”, as compared to those with mild dementia, had faster gait speed (mean difference 0.163 (95% CI: 0.053 to 0.273) *p*=0.004), shorter step time (-0.043 (-0.082 to -0.005) *p*=0.029), higher SPPB score (1.7 (0.5 to 2.8) *p*=0.005), and longer upright time (78.9 (18.9 to 139.0) *p*=0.011). The “no or questionable dementia” group, as compared to those with moderate to severe dementia, had faster gait speeds (0.208 (0.076 to 0.339) *p*=0.002) and higher SPPB score (1.8 (0.5 to 3.2) *p*=0.009). The estimated mean differences for step time and upright time variables were slightly smaller when comparing the “no or questionable dementia” group to those with moderate to severe dementia, and the evidence of a difference was weaker, in such a way that the reduction in step time and increase in upright time were not statistically significant at the 5% level.

**Table 3:** Results from linear regression analyses for gait characteristics, physical function and physical activity, on cognitive function, adjusted for age

	No or questionable dementia vs. Mild dementia Group 1 vs. Group 2		No or questionable dementia vs. Moderate to severe dementia Group 1 vs. Group 3		Mild dementia vs. Moderate to severe dementia Group 2 vs Group 3		Overall group effect
Characteristics	Mean difference (95% CI)	***P***-value	Mean difference (95% CI)	***P***-value	Mean difference (95% CI)	***P***-value	***P***-value
**Gait**							
Gait speed (m/s)	0.163 (0.053 to 0.273)	0.004	0.208 (0.076 to 0.339)	0.002	0.045 (-0.085 to 0.174)	0.496	0.002
Step time (s)	-0.043 (-0.082 to -0.005)	0.029	-0.035 (-0.082 to -0.011)	0.134	0.008 (-0.038 to 0.054)	0.730	0.001
Walk ratio, SL/Cad	0.03 (-0.01 to 0.08)	0.149	0.05 (-0.01 to 0.10)	0.111	0.01 (-0.04 to 0.07)	0.696	0.198
SD stride velocity (cm/s)	-0.209 (-0.819 to 0.401)	0.497	-0.070 (-0.795 to 0.655)	0.848	-0.139 (-0.578 to 0.856)	0.701	0.788
**Physical function**							
SPPB, score	1.7 (0.5 to 2.8)	0.005	1.8 (0.5 to 3.2)	0.009	0.2 (-1.1 to 1.5)	0.783	0.007
**Physical activity**							
Upright time, minutes	78.9 (18.9 to 139.0)	0.011	62.1 (-10.6 to 134.7)	0.093	-16.9 (-88.5 to 54.8)	0.641	0.031
Upright events, number	1.6 (-5.5 to 8.7)	0.661	2.1 (-6.5 to 10.7)	0.632	0.5 (-8.0 to 9.0)	0.906	0.862
Length of upright events, max	14.8 (-5.5 to 35.2)	0.151	16.0 (12.4, -8.6 to 40.7)	0.199	1.2 (-23.1 to 25.5)	0.922	0.271

Groups are based on the CDR score, where score 0 and 0.5 are group 1, score 1 is group 2, and score 2 and 3 are group 3. SPPB=The Short Physical Performance Battery. Physical activity was measured over a period of 2 to 7 days in this sample.

## Discussion

We quantified gait characteristics, physical function, and daily-life PA, in home-dwelling, older people assessed at a geriatric memory clinic. The patients were grouped according to severity of their dementia. Participants in the “no or questionable dementia” group had better physical function (higher SPPB scores) compared to participants with mild dementia and moderate to severe dementia. The “no or questionable dementia” group also had faster gait speed and shorter step time, and they were more physically active with longer upright time, as compared to the groups with dementia, although the evidence was stronger when compared to the moderate to severe than to the mild dementia group.

As decline in physical function, including gait, is shown to precede decline in cognitive function [31, 32], the results for the “no or questionable” dementia group, especially for gait speed with an average value of 1.1 m/sec, was somewhat higher than expected. However, this group is very heterogenous with a range in gait speeds from 0.5 to 1.5 m/sec, where there would be different reasons for why persons have lower gait speeds in this group. The same was found for physical function as measured by the SPPB, with mean value of 10.4, and a range from 5 to 12 in the “no or questionable dementia” group.

Slowing of gait speed is a prominent change of gait, and could be a strategy when gait becomes more challenging [33]. Gait speed is suggested to be included as a minimum measure regarding motor and cognitive changes, and in contrast to for example SPPB, would only be challenged with ceilings effect in high functioning individuals [34]. Other gait characteristics or qualitative aspects of gait, such as those included in this study, might be more relevant in earlier phases to describe early decline in gait. One example is the study from Hausdorff et al. (2018), where declines both in quantity and quality of gait where seen among patients with MCI who had fallen [35]. Even subtask evaluations of specific measures of physical performance could be relevant, as some changes probably occur early and reflect changes that are not yet clinically significant [36]. Although more advanced or technological-dependent testing could be relevant for specific research questions, gait speed is easy to measure and thus easy to implement in clinical evaluations [34].

Our results indicate a difference in physical behavior including gait and physical function, between those having no or questionable dementia and the mild stages of dementia. Our findings are congruent with a study showing that dementia patients were more sedentary and performed less PA as compared to cognitively healthy controls [37]. There could be different causes for these findings such as brain pathology (neurodegeneration and vascular) and physical function, environmental factors related to lack of opportunities for PA due to increasing apathy [38, 39] and/or lack of caregiver support [40].

Traditionally we assess cognitive and physical impairment separately. An integrated approach could contribute to better understanding of both cognitive and motoric impairments [4, 7, 41]. Including daily-life measurements could further provide important insight into how PA and physical function are related to cognitive impairment [42]. Integrated approaches seem to be important, as among others the combination of gait problems and cognitive impairments could for example increase the overall risk of falling [43]. A study from Australia including persons with dementia indicated an association between daily-life PA and cognitive function [44]. It could be important to prevent decline in daily-life PA for this group, especially if a person experience challenges with participation due to their cognitive impairment. More clinical studies are needed, so we can guide development of PA interventions especially targeted for persons with dementia. Interestingly, our results for the no or questionable dementia group showed close to 30 minutes more in upright as compared to reference data for 75-79 years old, community-based individuals without dementia [45], and the mild dementia group showed close to 50 minutes less spent upright. For our moderate/severe dementia group, with higher mean age, close to 30 minutes less time in upright was shown as compared to reference data for 80-84 years old individuals from the same sample [45]. In line with another recent study [11], strategies to improve healthy behaviors are needed, and we could speculate how to keep those with no or questionable dementia physically active as the potential to slower the a cognitive decline exist.

Acknowledging the increasing evidence for the associations between low levels of PA, sedentariness, and negative health outcomes [46], the follow-up of persons with dementia should include a focus on PA levels and physical functions. To be able to prevent decline in daily life functions, maintaining PA and physical function as long as possible is especially important for home-dwelling older adults [47]. With the increasing number of persons with age-associated dementia worldwide [48], and in the absence of a cure, healthcare professionals should ensure that people with dementia are encouraged to be physically active as this has the potential to delay functional decline [10]. Targeting high risk individuals for future dementia, with a focus on a dual decline in both memory and gait speed is suggested in recent studies [49, 50]. An evaluation of individuals gait, physical function and PA in the no or questionable dementia group is thus highly relevant, confirming a potential and a need for a wider focus in memory clinics.

There are some important limitations with this study. First, this is a cross-sectional study from one memory clinic with a relatively low number of participants. Due to a modest number of participants, we were not able to perform more complex analyses. Data on comorbidities that may impact gait, physical function and physical activity were not available in our sample. We also speculate that selection bias is present for our group 3, moderate to severe dementia, as one inclusion criterion was being home-dwelling, and more persons in this severity of dementia group probably have been admitted to a nursing home. If we compare our sample with data from the NorCog registry, where our participants also were included, the cognitive status of our moderate/severe dementia group could indicate that our clinic have included a more severe group of cognitive impaired individuals as compared to other memory clinics in Norway [51]. Our data provide the status at the time of diagnostic work-up. Although not being able to generalize, some important strengths should be highlighted. We included a relatively wide range of measures, including continuous registration of daily PA from body-worn sensors, performance-based measures of physical function and gait characteristics from gait-lab testing. The diagnostic evaluation was broad, ensuring important descriptive information of the sample included.

## Conclusion

We found that less severe dementia was associated with better gait function, better physical function, and higher levels of PA, adjusted for age. Physical measures could provide complementary information to cognitive measures, both for gait, physical function and PA, and should be considered as part of assessments in cognitive evaluations for example in memory clinics. Further studies should assess how measures of gait, physical function, and PA as complementary measures for cognitive status could improve clinical evaluations when suspecting cognitive declines. We also need to know how these additional and complementary measures of cognitive status can be used to guide interventions. Finally, we need more knowledge on which or what combination of measures are most useful in clinical practice and for what reasons.

## Data Availability

The datasets generated and analysed during the current study are not publicly available due to participant confidentiality

## Abbreviations

PA: Physical activity
CDR: Clinical dementia rating scale
SPPB: Short Physical Performance Battery
